# Proteome-Wide Analysis of Lysine 2-Hydroxyisobutyrylation in Candida albicans

**DOI:** 10.1128/mSystems.01129-20

**Published:** 2021-02-02

**Authors:** Hailin Zheng, Nana Song, Xiaowei Zhou, Huan Mei, Dongmei Li, Xiaofang Li, Weida Liu

**Affiliations:** a Department of Medical Mycology, Institute of Dermatology, Chinese Academy of Medical Science and Peking Union Medical College, Nanjing, Jiangsu, People’s Republic of China; b Center for Global Health, School of Public Health, Nanjing Medical University, Nanjing, China; c Department of Microbiology & Immunology, Georgetown University Medical Center, Washington, DC, USA; d Jiangsu Key Laboratory of Molecular Biology for Skin Diseases and STIs, Nanjing, Jiangsu, People’s Republic of China; NIAID, NIH

**Keywords:** posttranslational modification, lysine 2-hydroxyisobutyrylation, proteome, *Candida albicans*, posttranslational modification

## Abstract

Candida albicans is the most common human fungal pathogen, causing diseases ranging from mucosal to systemic infections for both immunocompetent and immunocompromised individuals. Lysine 2-hydroxyisobutyrylation is a highly conserved posttranslational modification found in a wide variety of organisms. In this study, we surveyed the biological impact of 2-hydroxyisobutyrylation on lysine residuals (Khib) in C. albicans. Using an antibody enrichment approach along with the traditional liquid chromatography-tandem mass spectrometry (LC-MS/MS) method, we analyzed the pattern of Khib-modified proteins and sites in one wild-type strain of C. albicans. We identified 1,438 Khib-modified proteins with 6,659 modified sites in this strain, and a more detailed bioinformatics analysis indicated that the Khib-modified proteins are involved in a wide range of cellular functions with diverse subcellular locations. Functional enrichment analysis featured several prominent functional pathways, including ribosome, biosynthesis of antibiotics, biosynthesis of secondary metabolites, biosynthesis of amino acids and carbon metabolism, of which the ribosome pathway is the most affected pathway. Even compared with the reported numbers of lysine acetylation (Kac) and succinylation (Ksuc) sites, the numbers of Khib-modified sites on ribosomal proteins remained the highest for C. albicans. These bioinformatic results suggest that 2-hydroxyisobutyrylation may play an indispensable role in the regulation of the ribosomal biogenesis and protein translation. Findings in this study may provide new insights for studying posttranslational modification (PTM)-associated mechanisms in fungal development and pathogenicity.

**IMPORTANCE** C. albicans is one of the most commonly reported fungal pathogens in mucosal and systemic infections. A better understanding of its growth habits and metabolic processes in the host should help improve defense strategies. The newly discovered protein posttranslational modification (PTM) on histones is one epigenetic mechanism which has been linked to many pathogenic events, including cancers. The types of PTM and their pathogenic roles in C. albicans are still somewhat poorly understood, even though studies of C. albicans based on acetylation inhibitors have shed some light on their function, and it seems that PTMs regulate pathogenic adhesion factors. Here, we quantified and analyzed the occurrence of lysine 2-hydroxyisobutyrylation (Khib) in C. albicans. The Khib-modified proteins are enriched with respect to carbon metabolism, ribosomal biogenesis, and protein translation in C. albicans.

## Abstract (precis)

C. albicans is one of the most commonly reported fungal pathogens in mucosal and systemic infections. A better understanding of its growth habits and metabolic processes in the host should help improve defense strategies.

## INTRODUCTION

Candida albicans is the most common human fungal pathogen and causes diseases ranging from mucosal inflammations to systemic infections. It often appears asymptomatically, colonizing mucosal surfaces in healthy hosts as a commensal; however, any disruption of the host immune system promotes its proliferation and invasion of virtually any site of the body (1). For life-threatening systemic and bloodstream infections such as are often found in immunocompromised hosts, the mortality rate can be as high as 50%. Today, candidiasis is also a common cause of nosocomial infections in clinical settings. Currently, effective drugs of choice against C. albicans remain limited, and some are quite cytotoxic. The emergence of resistance to azoles and echinocandins further complicates antifungal treatment (2). Therefore, novel drug targets are essential but require an understanding of fungal biology.

The genome of C. albicans is ∼16 Mb, encoding more than 14,000 proteins, of which about 7,600 proteins are expected to be functionally (3) expressed at different stages of development in various quantities. In order to respond to environmental changes, living organisms need to gain the ability to adapt to and survive in unfavorable environments. During this adaptation, certain new proteins must be synthesized in certain amounts; however, protein posttranslational modification (PTM)—a dynamic and reversible process—may occur much faster than the protein synthesis, which allows a faster response to environmental challenges. In recent years, the roles of PTM of histone lysine residues in metabolic regulation, protein interaction, cell signaling, and other biological processes have become increasingly evident (4, 5). PTM is a process in which a chemical moiety is covalently added to certain amino acid groups after a protein is translated from its mRNA template (6). The common types of PTM include glycosylation, ubiquitination, nitrosylation, methylation, phosphorylation, lipidation, acetylation, succinylation, and crotonylation, all of which play important roles in cell biology and pathogenesis (5, 7–13).

2-Hydroxyisobutyrylation is a newly discovered PTM type, and the addition of elemental composition (C_4_H_7_O_2_) gives a mass shift of +86.0368 Da (or a multiple thereof) under mass spectrometry analysis. The 2-hydroxyisobutyrylation of lysine (Khib) was first reported in active histone in 2014 (14) and was later found not only in HeLa cells but also in mouse embryonic fibroblast cells, *Drosophila* S2 cells, and cells of the yeast Saccharomyces cerevisiae. These findings proved that Khib is an evolutionarily conserved and dynamic marker in eukaryotic cells (14–18). However, systematic proteome analysis of Khib in other species has also proven its existence in plants (19, 20) and in bacteria (21). In previous studies, the observations that CobB can catalyze Khib in prokaryotes *in vivo* and P300 can catalyze Khib in mammalian cells were confirmed (22, 23). From a biochemical point of view, this modification on the genome-associated histones would neutralize the positive charge of the lysine on the histones and a hydroxyl group would enable the modified lysine to form hydrogen bonds with other molecules. Indeed, this hydroxyl group is known to be important for the regulatory roles of certain protein. For example, it has been associated with hypoxia-inducible factor α subunit (HIFα) (24), which is a key transcription factor in the mammalian response to oxygen deficiency. The wide distribution of Khib in histones has been proven to affect starch biosynthesis, glycolysis/gluconeogenesis, lipid metabolism, the tricarboxylic acid (TCA) cycle, protein biosynthesis, and protein processing in the cells. The dysfunction of Khib has also been associated with pathogenesis of some diseases such as bladder cancer (25).

The pathogenetic effects of posttranslational modifications in C. albicans have not been widely studied apart from limited surveys of activities such as phosphorylation, acetylation, and succinylation (26–28). To better understand the mechanisms of PTMs and their impact on cell function in C. albicans, we performed a large-scale analysis of lysine 2-hydroxyisobutyrylation on C. albicans using an enrichment of Khib-modified peptides and the liquid chromatography-tandem mass spectrometry (LC-MS/MS) method.

## RESULTS

### Khib is highly abundant in C. albicans histones.

**FIG 1:**
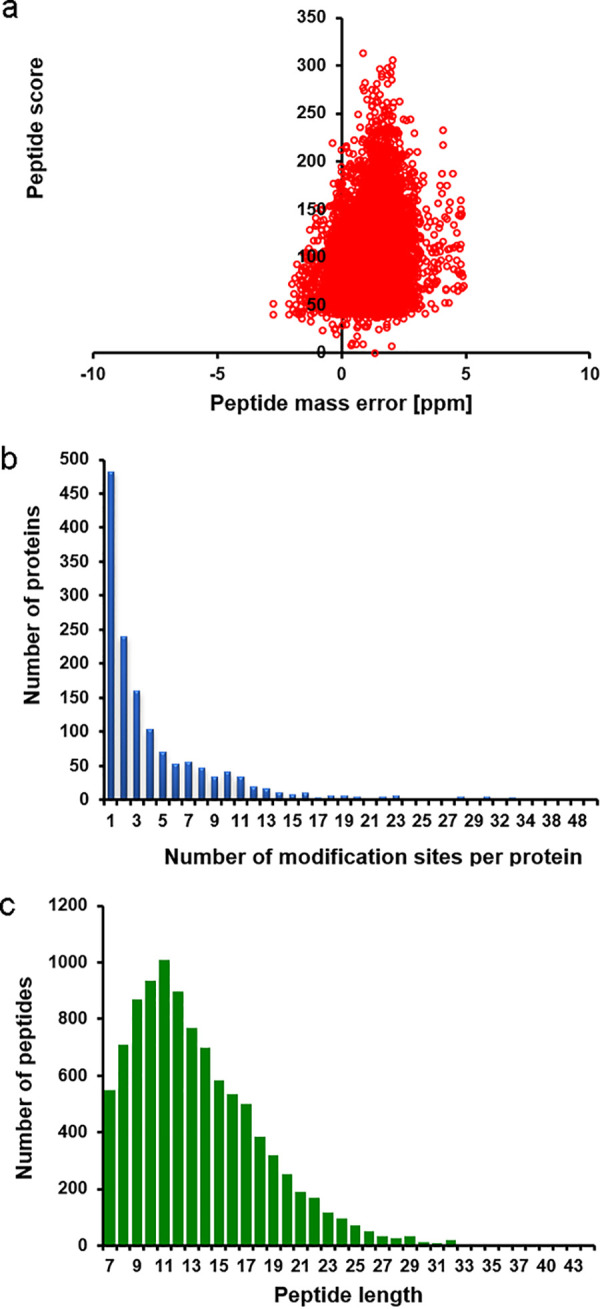
QC validation of MS data. (a) Mass error distribution of all identified peptides. (b) Number of modification sites per protein. (c) Peptide length distribution.

In this study, the analysis of all 2-hydroxyisobutyrylated proteins of C. albicans was carried out after enrichment with 2-hydroxyisobutyrylation-specific antibody. We observed a large number of protein bands occupying a wide protein mass range within the LC-MS/MS spectrum (Fig. 1a). When mass errors were quantified, the distribution was near zero, with most of them having values of <10 ppm, indicating that the data were of good quality. The distribution of modified sites indicated that 482 proteins contained only 1 site of Khib modification; however, a very low percentage contained more than 20 Khib sites (Fig. 1b). Since trypsin was used to digest the protein samples, 8,484 of the identified peptides exhibited lengths between 8 and 20 amino acids (aa), with a peak length of 11 aa (Fig. 1c). These peptides were traceable to only 1,438 proteins with a total of 6,659 Khib sites (see Data Set S1 in the supplemental material), which accounted for 9.8% of the total proteins of C. albicans (1,438/1,4633) (3). To understand the overall distribution of 2-hydroxybutyrylation in C. albicans, we compared the number of 2-hydroxybutyrylated proteins that were identified in this study with those reported for other prokaryotes and for eukaryotes such as budding yeast (S. cerevisiae), the nonseed plant Physcomitrella patens, human cervical cancer (HeLa) cells, and rice (Oryza sativa) seeds. The results shown in Table 1 reveal significant degrees of 2-hydroxybutyrylation among species with regard to both numbers of modified sites and proteins modified. Whereas the average number of 2-hydroxybutyrylation sites for each protein reaches 4.6 in C. albicans, it reaches 3.9 in S. cerevisiae (as measured over 365 proteins), 3.99 in *P. patens* (over 3,000 proteins), 3.7 in human cells (over 1,681 proteins), and 3.9 in *R. seeds* (over 2,498 proteins) (29). Apparently, C. albicans possesses the highest level of 2-hydroxybutyrylation per protein of all these species.

**TABLE 1:** Comparison of 2-hydroxyisobutyrylation sites and 2-hydroxyisobutyrylated proteins in C. albicans and other organisms

Species	No. of:		Reference
	Sites	Proteins	
C. albicans	6,659	1,438	This study
S. cerevisiae	1,449	365	29
*P. patens*	11,970	3,000	29
Homo sapiens (HeLa cells)	6,231	1,681	29
*O. sativa* (rice seeds)	9,818	2,498	29

10.1128/mSystems.01129-20.1 DATA SET S1 Protein and functional annotation. Download Data Set S1, XLSX file, 1.7 MB. Copyright © 2021 Zheng et al. 2021 Zheng et al. This content is distributed under the terms of the Creative Commons Attribution 4.0 International license.

### Motif analysis of the identified modified peptides.

**FIG 2:**
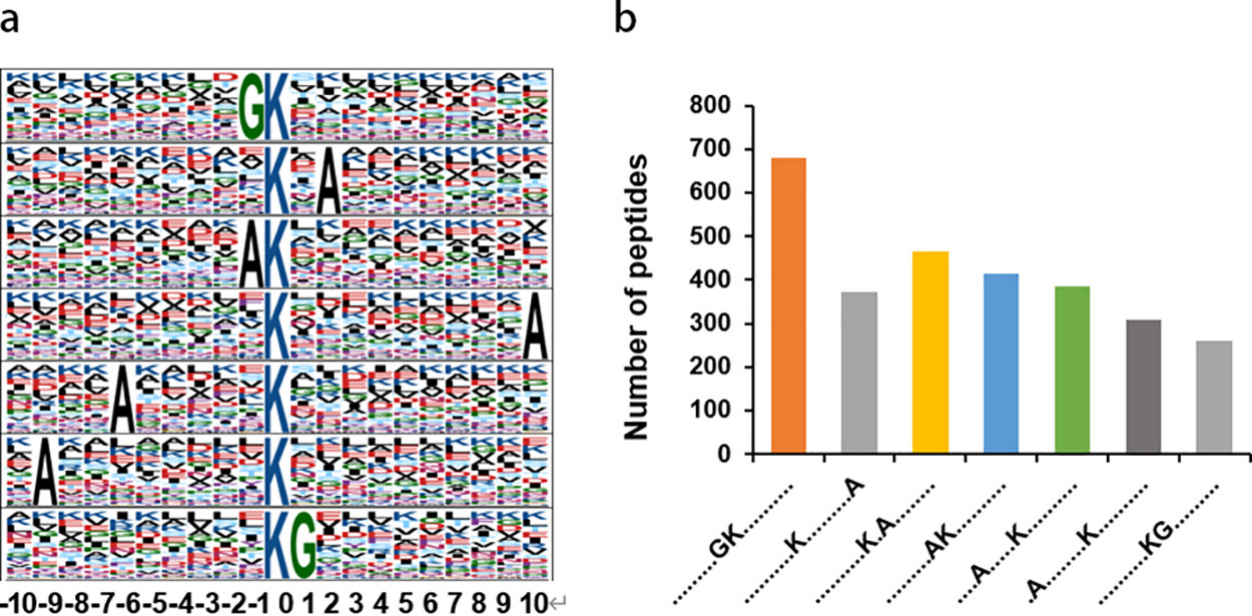
Sequence properties of the 2-hydroxybutyrylated peptides. (a) 2-Hydroxybutyrylation motifs identified by motif-x. The height of each letter corresponds to the frequency of that amino acid residue in that position. The central K refers to the 2-hydroxybutyrylated lysine. (b) Number of identified peptides containing 2-hydroxybutyrylated lysine in each motif.

In order to characterize the nature of the 2-hydroxybutyrylation process in C. albicans, the sequence motifs of 2-hydroxybutyrylated lysine in all of the 2-hydroxybutyrylated sites were analyzed using the program motif-x, a software tool that was designed to extract overrepresented patterns in any given set of sequences. Of all identified Khib peptides, 4,601 were found to include the desired amino acid sequence from the 10 amino acids on either side of the 2-hydroxybutyrylated lysine. The analysis of 2-hydroxybutyrylated sites in these peptides resulted in seven motifs with different abundances: GK^2-hy^, K^2-hy^*A, A**K^2-hy^, K^2-hy^*********A, A*****K^2-hy^, A********K^2-hy^, and K^2-hy^G (Fig. 2a), where K^2-hy^ marks the 2-hydroxybutyrylated lysine and each asterisk indicates a single amino acid residue. Among these motifs, GK^2-hy^ in Fig. 2b is strikingly abundant, covering approximately 18% of all the 2-hydroxybutyrylated peptides we identified, which may suggest its susceptibility to 2-hydroxybutyrylation in C. albicans. In addition, the amino acids around Lys (K) in these motifs seem to prominently feature hydrophobic alanine (A) (66.33%) and neutral glycine (G) (Fig. 2b). In contrast, Lys (K) with positively charged amino acids and hydrophobic Leu (L) predominates in other species (S. cerevisiae, *P. patens*, human HeLa cells, and *O. sativa* seeds) (29).

### Functional classification of Khib-modified proteins.

**FIG 3:**
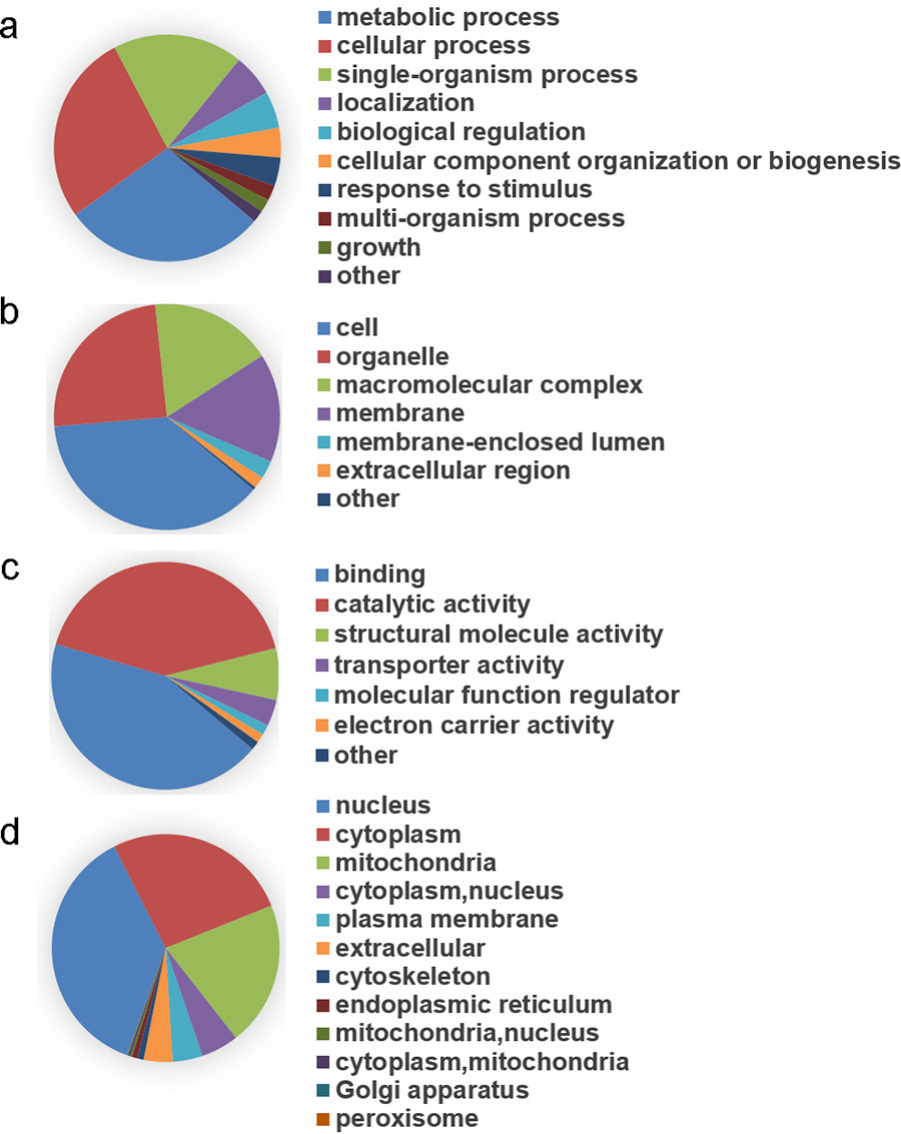
GO functional classification and subcellular location of Khib-modified proteins. Functional classification of 2-hydroxybutyrylated proteins according to GO annotation information were divided into biological processes (a), cellular components (b), molecular functions (c), and subcellular localization of Khib proteins (d) according to WoLF PSORT software.

All 2-hydroxyisobutyrylation-associated proteins that we identified from C. albicans were annotated via Gene Ontology (GO) functional classification. The annotation indicated that Khib-modified proteins predominantly participate in metabolism (29%), cellular processes (27%), and single-organism processes (19%) in the biological process category (Fig. 3a). Furthermore, in terms of the GO cellular component category, the majority of modified proteins are distributed in cells (37%), organelles (25%), and the macromolecular complex (18%) (Fig. 3b). With regard to the GO molecular function category, proteins modified by Khib are mainly enriched in two respects: binding (43%) and catalytic activity (42%) (Fig. 3c). The subcellular localization prediction using WoLF PSORT software showed a generally wide distribution of Khib-modified proteins, in which nuclear proteins contain the largest portion of Khib-modified proteins (37%), followed by the cytoplasm (27%) and finally mitochondria (21%) (Fig. 3d). Functional characterization of these modified proteins suggested that they commonly regulate pathways involved in translation, ribosomal structure, and biogenesis in C. albicans.

### Functional enrichment analysis of Khib proteins.

**FIG 4:**
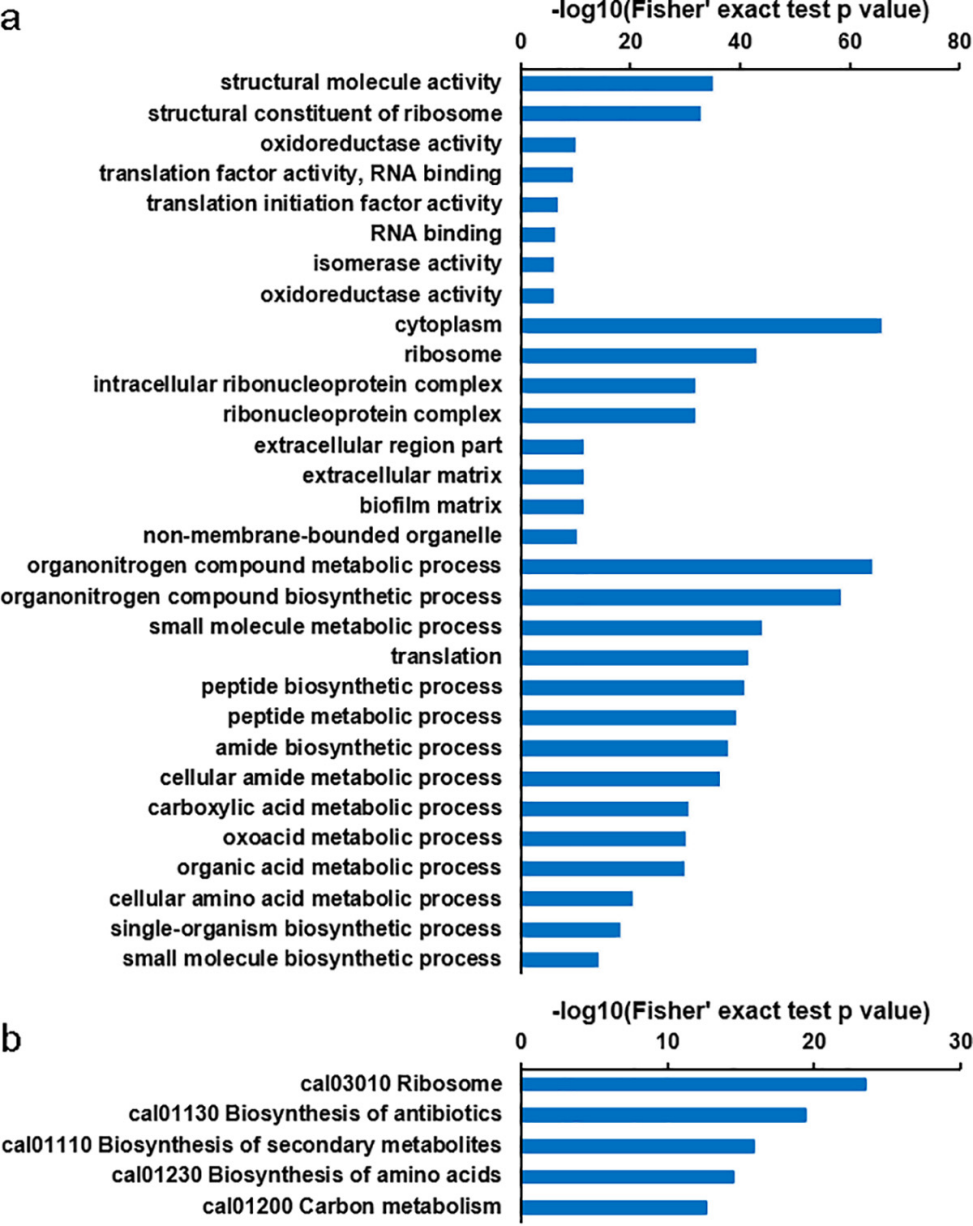
Enrichment analysis of the 2-hydroxybutyrylated proteins in C. albicans. (a) Enrichment analysis of the 2-hydroxybutyrylated proteins based on the classification of GO annotation in terms of biological process, cellular component, and molecular function. (b) KEGG pathway enrichment analysis.

Through GO enrichment analysis, functional predication revealed that 2-hydroxyisobutyrylation in C. albicans was significantly enriched in ribosome biogenesis and in cytoplasm by referring to the structural constituents of ribosome, oxidoreductase activity in organonitrogen compound metabolic processes, organonitrogen compound biosynthetic processes, and small-molecule metabolic processes (Fig. 4a). In KEGG pathway enrichment analysis, a total of five significantly enriched pathways were revealed in modified proteins, specifically ribosomal biogenesis, biosynthesis of antibiotics, biosynthesis of secondary metabolites, biosynthesis of amino acids, and carbon metabolism (Fig. 4b). Obviously, the ribosome pathway was the most enriched pathway in these modified proteins.

### Overlap of lysine 2-hydroxyisobutyrylation with other lysine modifications.

PTM data such as phosphorylation, malonylation, acetylation, crotonylation and succinylation, etc., are all good bases for our understanding of the biological functions of PTMs in prokaryotes and eukaryotes. Currently, 469 quantitative proteomics of PTMs have been stored in the UniProt database, of which 326 are from eukaryotes (9) and which even include a few sets from C. albicans (26–28). With the availability of the phosphoproteome, acetylateome, and succinylateome of C. albicans, we have made a preliminary comparative analysis among acetylation, succinylation, and 2-hydroxyisobutyrylation in C. albicans due to the occurrence of all three PTMs on lysine residuals. Notably, the number of proteins with any single site of modification (whether acetylation, succinylation, or 2-hydroxyisobutyrylation) is much lower than that of proteins showing up under multiple sites of one modification, indicating a complex of integration of these PTMs on a certain biological process in this organism.

**FIG 5:**
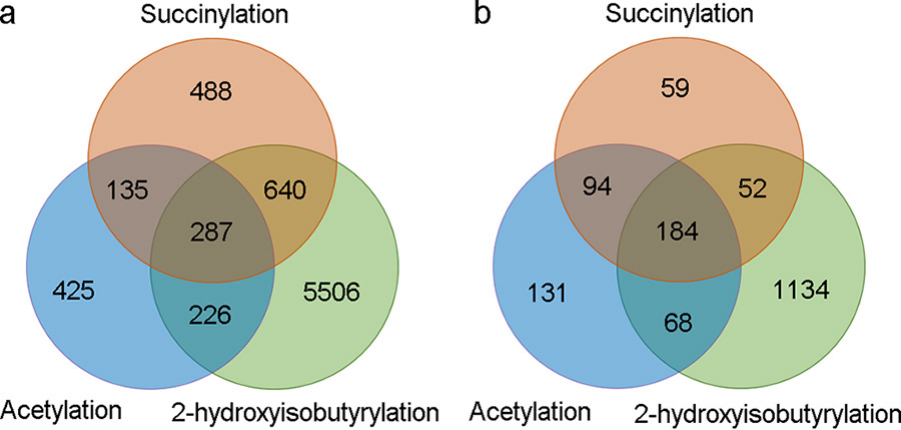
Venn diagrams showing the overlap of 2-hydroxybutyrylation, acetylation, and succinylation based on modification sites (a) and modified proteins (b).

Further, an overlap analysis of all three types of PTM was carried out, and the results are shown in Data Sets S2 and S3. Generally, the modification level of lysine 2-hydroxyisobutyrylation was significantly higher than that of acetylation or succinylation in C. albicans (Fig. 5). When the results are viewed from the standpoint of the Khib-modified sites (and not the proteins), there are 287 lysine sites showing all three types of modification at these sites (Fig. 5a). Also, the overlap of 2-hydroxyisobutyrylation with succinylation seems to be more common than the overlap of 2-hydroxyisobutyrylation with acetylation, but this is probably related to the much smaller number of modification sites in acetylation. From the standpoint of Khib-modified proteins, a total of 184 proteins were able to undergo all three modifications simultaneously (Fig. 5b). On the other hand, 2-hydroxyisobutyrylation alone seems to be found in more proteins (1,134) than either succinylation (59 proteins) or acetylation (131 proteins).

### 2-Hydroxyisobutyrylation of ribosome proteins in C. albicans.

The ribosome substrates aminoacyl tRNAs and mRNAs interact with rRNA to produce polypeptides (30). As shown in the KEGG enrichment analysis, a total of 707 Khib sites in ribosomal proteins were identified in C. albicans, a much higher number than the number of lysine modification sites with succinylation (247) or acetylation (117) (Data Set S4).

10.1128/mSystems.01129-20.2 DATA SET S2 Comparison with other modifications of Khib sites. Download Data Set S2, XLSX file, 0.9 MB. Copyright © 2021 Zheng et al. 2021 Zheng et al. This content is distributed under the terms of the Creative Commons Attribution 4.0 International license.

10.1128/mSystems.01129-20.3 DATA SET S3 Comparison with other modifications of Khib proteins. Download Data Set S3, XLSX file, 0.9 MB. Copyright © 2021 Zheng et al. 2021 Zheng et al. This content is distributed under the terms of the Creative Commons Attribution 4.0 International license.

10.1128/mSystems.01129-20.4 DATA SET S4 Ribosomal proteins of three modifications. Download Data Set S4, XLSX file, 1.3 MB. Copyright © 2021 Zheng et al. 2021 Zheng et al. This content is distributed under the terms of the Creative Commons Attribution 4.0 International license.

**FIG 6:**
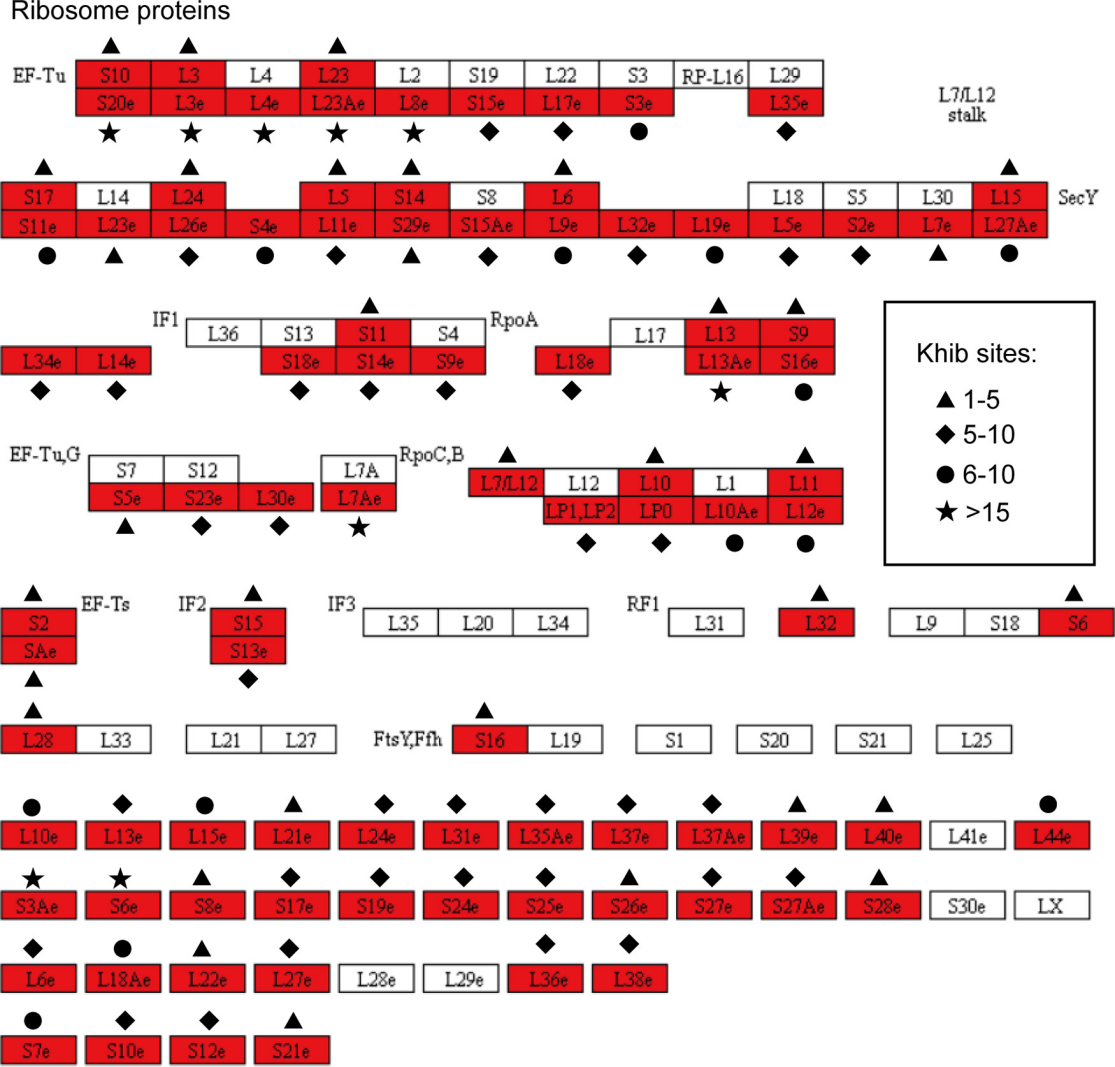
The 2-hydroxyisobutyrylated ribosomal proteins in C. albicans. The identified 2-hydroxyisobutyrylated proteins are colored. Different markers represent different numbers of modification sites. Khib, lysine 2-hydroxyisobutyrylation.

Elongation factor Tu (EF-Tu) and SelB are translational GTPases that deliver aminoacyl-tRNAs (aa-tRNAs) to the ribosome. In each canonical round of elongation during translation, aa-tRNA, assisted by EF-Tu, decodes mRNA codons and brings the respective amino acid to the growing peptide chain (31). We found that proteins in the EF-Tu protein family are highly modified by 2-hydroxyisobutyrylation. For example, five proteins exhibit more than 15 modified sites, and three proteins have more than 20 modified sites (Fig. 6). A similar phenomenon was also shown in L7/L12 stalk proteins. The dimers of L7/L12 consist of the 1030–1124 region of the 23S rRNA ribosomal proteins L10 and L11. L7 has the same sequence as L12 with the addition of an *N*-terminal acetylation (32). The L7/L12 stalk is associated with translation initiation, elongation, and termination in bacterial ribosomes (the 70S ribosome) and is a general morphological feature of the large subunits of representative prokaryotes (33). Also, the GTPase activity of EF-G requires the presence of L7/L12, which is critical for ribosomal translocation (32). In this study, we identified a total of 53 Khib-modified sites on L10, L11, and L7/L12. These data suggest that the function of the L7/L12 stalk and EF-Tu are largely regulated by lysine 2-hydroxyisobutyrylation in C. albicans.

## DISCUSSION

The newly discovered protein posttranslational modification lysine 2-hydroxyisobutyrylation is one protein PTM that has been found to occur in a wide variety of organisms. Recently, studies on PTMs have been extended from histone to nonhistone proteins in a variety of organisms, including humans and plants (14, 21, 34). In this study, we use quantitative proteomics to characterize this lysine in C. albicans. A total of 1,438 proteins featuring a total of 6,659 Khib sites were found, and the Khib modification appears to be most pronounced in this organism in the areas of metabolic regulation, ribosomal biogenesis, and various biological processes.

The impact of Khib modification on the regulation of cellular processes is well known, but the mechanisms of individual Khib modifications remain undiscovered in C. albicans. However, one may hope that some preliminary studies on other organisms might provide a good reference to understand Khib regulation in C. albicans. For example, the concentration of H4K8hib was found to fluctuate in response to the availability of carbon in S. cerevisiae, with low-glucose conditions leading conversely to diminished H4K8hib (16). Protein expression at different levels with different modification patterns (including Khib) are also associated with parasitic phenotypes of Toxoplasma gondii (35). A study on prokaryotes has dissected a Khib-mediated molecular mechanism for the regulation of bacterial growth as well as the activity of metabolic enzymes (22). Functional annotation analysis of Oryza sativa has suggested that a wide variety of vital biological processes are preferably regulated by Khib, including carbon metabolism, lipid metabolism, and protein biosynthesis (19). Khib modification has even been associated with some diseases. In a comparative survey of Khib proteomics between lesional psoriasis and healthy skin, the authors found that 94 sites in 72 proteins found in lesional skin tissues were upregulated, while another 51 sites from 44 proteins were downregulated (18). Among the differentially expressed proteins, the phosphatidylinositol 3-kinase (PI3K)-Akt signaling pathway was prominent in psoriatic skin, suggesting that Khib plays a role in psoriatic pathogenesis. Based on the functional annotation analysis, we find that the data for C. albicans in this study seem quite similar to the data for certain other organisms studied elsewhere by similar techniques, suggesting that Khib modification plays a role in carbon metabolism and ribosomal biogenesis in C. albicans.

Furthermore, an overlap analysis of acetylation, succinylation, and 2-hydroxyisobutyrylation was carried out, and there are 287 lysine sites and 184 proteins that undergo all three modifications. The results suggested that there is some correlation between these three modifications. The presence of the P300 may explain some of this. P300, a well-studied transcription coactivator, has emerged as the most promiscuous acyl-transferase to date. Beyond its originally described acetyltransferase activity, p300 can also catalyze histone propionylation, butyrylation, 2-hydroxyisobutyrylation, succinylation, and crotonylation (23, 36, 37).

Ribosomes are molecular machines that are highly regulated during biochemical processes. In eukaryotic cells, 80S ribosome consists of a large 60S subunit and a small 40S subunit (38). Obviously, the function of protein translation is so important for pathogenic-organism growth in a host that it is often favored as a target for antibiotic discovery. Also, the relatively small ribosome in bacteria further promotes the efficacy of these antibiotics (39). We found a total of 707 Khib-modified sites among the constituents of the ribosomal structure and in EF-Tu for protein translation, indicating that this modification is a major regulatory mechanism in C. albicans ribosome biogenesis. Whether the regulatory function of the Khib-modified proteins can be directed to inhibition of ribosomal biogenesis in C. albicans or its growth in the host remains to be studied.

In conclusion, we completed the first analysis of 2-hydroxyisobutyrylation in the human pathogen C. albicans using a series of highly sensitive proteomic methods. Our findings broaden the current perception of the involvement of 2-hydroxyisobutyrylation in protein modification and illustrate the possible functions and regulatory mechanisms of this newly identified PTM in C. albicans. The results provide new insights for studying PTM-associated mechanisms in fungal development and pathogenicity.

## MATERIALS AND METHODS

### Strains and culture.

C. albicans strain SC5314 was grown at 28°C on a rotary shaker (220 rpm) overnight in yeast extract-peptone-dextrose (YPD) medium and used as stock cultures. Ten milliliters of stock culture was inoculated into flasks containing 100 ml of fresh YPD medium and cultured with shaking (220 rpm) at 28°C for 4 h until the optical density at 600 nm (OD_600_) reached 0.8.

### Protein extraction and digestion.

The cultured cells were harvested by centrifugation at 6,000 rpm and 4°C for 10 min and were then washed twice with 4°C phosphate-buffered saline (PBS). Then sample was ground in liquid nitrogen into powder and then transferred to a 5-ml centrifuge tube. Four volumes of lysis buffer (8 M urea, 1% Triton-100, 10 mM dithiothreitol, 1% protease inhibitor cocktail [100 mM AEBSF {4-(2-aminoethyl)benzenesulfonyl fluoride hydrochloride}, 1.5 mM E-64, protease inhibitor, 2 mM pepstatin A, and 100 mM EDTA {Calbiochem}], 3 μM trichostatin A [TSA; Sigma], 50 mM nicotinamide [NAM; Sigma], and 2 mM EDTA) was added to the cell powder, followed by sonication three times on ice using a high-intensity ultrasonic processor (Scientz). The remaining debris was removed by centrifugation at 20,000 × *g* at 4°C for 10 min. Finally, the protein was precipitated with cold 20% trichloroacetic acid for 2 h at 4°C. After centrifugation at 12,000 × *g* and 4°C for 3 min, the supernatant was discarded. The remaining precipitate was washed with cold acetone three times. The protein was redissolved in 8 M urea and the protein concentration was determined with a bicinchoninic acid (BCA) assay kit (Beyotime) according to the manufacturer’s instructions. For digestion, the protein solution was reduced with 5 mM dithiothreitol for 30 min at 56°C and alkylated with 11 mM iodoacetamide for 15 min at room temperature in dark. The protein sample was then diluted by adding 100 mM NH_4_HCO_3_ until the urea concentration dropped below 2 M. Finally, trypsin was added at 1:50 (trypsin-to-protein mass ratio) for the first digestion overnight and 1:100 (trypsin-to-protein mass ratio) for a second 4-h digestion.

### Enrichment of lysine 2-hydroxyisobutyrylated peptides.

To enrich Khib-modified peptides, tryptic peptides dissolved in NETN buffer (100 mM NaCl, 1 mM EDTA, 50 mM Tris-HCl, 0.5% NP-40 [pH 8.0]) were incubated with prewashed antibody beads (lot number PTM-804; PTM Bio) at 4°C overnight with gentle shaking. Then the beads were washed four times with NETN buffer and twice with H_2_O. The bound peptides were eluted from the beads with 0.1% trifluoroacetic acid. Finally, six eluates were combined and vacuum dried. For LC-MS/MS analysis, the resulting peptides were desalted with Ziptips C_18_ resin pipette tips (Millipore) according to the manufacturer’s instructions.

### Qualitative proteomic analysis by LC-MS/MS.

The tryptic peptides were dissolved in 0.1% formic acid (solvent A), directly loaded onto an in-house-made reversed-phase analytical column (15-cm length, 75-μm inner diameter). The gradient comprised an increase from 6% to 23% solvent B (0.1% formic acid in 98% acetonitrile) over 36 min, an increase from 23% to 35% over 8 min, and an increase to 80% over 3 min, followed by holding at 80% for the last 3 min, all at a constant flow rate of 700 nl/min on an EASY-nLC 1000 ultraperformance liquid chromatography (UPLC) system.

The peptides were subjected to nanospray ionization (NSI) followed by tandem mass spectrometry (MS/MS) in Q Exactive Plus (Thermo) coupled online to the UPLC. The electrospray voltage applied was 2.0 kV. The *m/z* scan range was 350 to 1,600 for a full scan, and intact peptides were detected in an Orbitrap instrument at a resolution of 60,000. Peptides were then selected for MS/MS using the normalized collision energy (NCE) setting 28, and the fragments were detected in the Orbitrap at a resolution of 15,000. The data-dependent procedure alternated between one MS scan and 20 MS/MS scans with 15.0 s dynamic exclusion. The automatic gain control (AGC) was set at 1E5.

### Database search.

MaxQuant integrated with the Andromeda search engine (v.1.5.2.8) was used to process the MS/MS data (40). The tandem mass spectra were searched against the UniProt C. albicans strain_SC5314 database (2018.05 version) concatenated with the reverse decoy database. Trypsin/P was designated a lyase, and up to 4 missing cuts were allowed. The precursor ion mass errors in First search and Main search were set to 20 ppm and 5 ppm, respectively, and the secondary fragment ion mass error was set to 0.02 Da. Carbamidomethylation on cysteine was specified as the fixed modification, and 2-hydroxyisobutyrylation on lysine, acetylation on protein N-term, and oxidation on Met were specified as variable modifications. The false discovery rate (FDR) thresholds for proteins, peptides, and Khib sites were set to 1%.

### GO annotation.

Gene Ontology (GO) is a major bioinformatics initiative to unify the representation of gene and gene product attributes across all species. The proteome of GO annotation was derived from the UniProt Gene Ontology Annotation (GOA) database (http://www.ebi.ac.uk/GOA/). All identified proteins were first to converted to UniProt ID and then mapped under GOA IDs. When identified proteins were not annotated by the UniProt-GOA database, InterProScan software was used to annotate functions of proteins based on protein sequence alignment method. Gene Ontology annotation analysis was based on three categories: biological process, cellular component, and molecular function.

### KEGG pathway annotation.

The Kyoto Encyclopedia of Genes and Genomes (KEGG) connects known information on molecular interaction networks, including pathways and complexes (the pathway database), genome projects (including the gene database), and biochemical compounds and reactions (including compound and reaction databases). The KEGG database was used to annotate protein pathways found in this study using KEGG online service tools, i.e., the KEGG Automatic Annotation Server (KAAS) KEGG mapper.

### KOG annotation.

The Eukaryotic Orthologous Groups (KOG) annotation of the proteome was derived from the NCBI-COG database (https://www.ncbi.nlm.nih.gov/COG/). The sequences of differentially modified proteins were searched in Basic Local Alignment Search Tool (BLAST) version 2.2.28 to obtain protein KOG annotation.

### Subcellular localization.

The eukaryotic cells are elaborately subdivided into functionally distinct membrane-bound compartments. Major constituents of these compartments are extracellular space, cytoplasm, nucleus, mitochondria, Golgi apparatus, endoplasmic reticulum (ER), peroxisome, vacuoles, cytoskeleton, nucleoplasm, nucleolus, nuclear matrix, and ribosomes. WoLF PSORT is an updated version of PSORT/PSORT II for the prediction of eukaryotic sequences (41). We used this software to predicate subcellular localizations of identified proteins.

### Motif analysis.

The software motif-x was used to identify the context sequences of amino acids around 2-hydroxyisobutyrylated lysine residues (10 amino acids upstream and downstream of the site) in all protein sequences (42). The database search for these protein sequences used default parameters in each database. When the number of peptides in a certain characteristic sequence form is greater than 20 and *P* is less than 0.000001, the sequence is considered a motif of the modified peptide.

### Functional enrichment and statistic analysis.

For each category of GO annotation, a two-tailed Fisher’s exact test was employed to test the enrichment of the modified protein against all proteins databases. The GO with a corrected *P* value of <0.05 is considered significant. The same two-tailed Fisher’s exact test and a corrected *P* value of <0.05 were also considered significant in KEGG for pathway analysis and InterPro for protein domain analysis.

### Data availability.

The mass spectrometry proteomics data have been deposited in the ProteomeXchange Consortium via the PRIDE partner repository with the data set identifier PXD023013.
